# Does the location of a narrative comment section affect feedback on a lecture evaluation form?

**DOI:** 10.5116/ijme.58e2.96c3

**Published:** 2017-04-21

**Authors:** Joseph R. Pare, Abbas H. Kothari, Jeffrey I. Schneider, Gabrielle A. Jacquet

**Affiliations:** 1 Department of Emergency Medicine, Boston Medical Center, Boston, MA, USA

**Keywords:** Narrative comment, feedback, lecture evaluation form

## Introduction

Although the structure and composition of graduate medical education (GME) is evolving with medical and technological advances, didactic lecture format instruction remains a critical component in many training environments internationally. In the US, the Accreditation Council for Graduate Medical Education (ACGME) publishes program requirements outlining educational guidelines for 133 specialties and subspecialties.^1^ These requirements mandate Emergency Medicine (EM) residency programs must have an average of at least five hours of dedicated instruction per week with 50% of these hours given by faculty members. Trainees therefore often lead a significant portion of didactic sessions. Developing presentation skills is a critical talent to learn, as junior physicians become educators. Additionally, US accreditation bodies require evaluations to measure participation and educational effectiveness of these didactic presentations. Learner evaluations are one mechanism used to meet this requirement.

Formative assessment and feedback have been shown to change trainees’ behavior.^2^^-^^5^ Recent literature also supports the notion that trainees do not benefit from feedback in the form of numerical marks but rather from narrative, specific feedback that explicitly states the areas that require improvement.^2^^,^^3^^,^^6^ Despite this evidence, there is little data about the components of an effective evaluation tool. One common example, a numerical Likert scale has been shown to be less helpful than narrative or verbal feedback.^7^ To our knowledge, there is no validated or recommended tool to measure educational effectiveness while also providing meaningful feedback to resident lecturers.

The authors sought to test the hypothesis that by relocating a blank space for written comments from the bottom of a lecture evaluation form to the top, it would convey the importance of narrative feedback to the evaluator, motivate the evaluator to provide feedback more frequently, and increase the quality of feedback provided to our resident lecturers. The purpose of this study was to determine if moving the narrative feedback section on a lecture evaluation form from the bottom to the top of the form increases the presence and the quality of written narrative feedback.

### Implementation of a new lecture evaluation form

A group of faculty members and residents developed a novel lecture evaluation form that included both Likert numerical scoring assessments and a large blank space for hand-written comments. An iterative process was used to develop a feedback form that was easy to use while also providing meaningful instruction and guidance to those delivering educational sessions. The open response/blank space on the form was separated into two columns labeled “Strengths” and “Areas for Improvement”. Two versions of this form were created – they were identical except for one had a comments section at the top of the page, above the Likert questions, and the other had the comments section below the Likert questions. For comparison and analysis, the form with the comment section at the bottom of the page was the reference form, while the one with the feedback space at the top was the test form. The authors’ Institutional Review Board deemed the study exempt as an educational improvement study that did not include patient information or identifiers.

Evaluation forms were randomly distributed to faculty, residents, and medical students in the audience during our weekly lecture-based conference. Participants were blinded to the objectives of the study, and forms completed by the study authors were excluded from analysis. Forms were collected and analyzed by two independent resident reviewers who evaluated the forms for the presence of written comments and the value of each comment.

The usefulness of each comment was assessed by the reviewers based a 0-3 ordinal scale.^7^ Zero points were given if no comment was present; one point was assigned for a non-specific comment (e.g. “great lecture”); two points were awarded for a moderately specific comment that identified an area for improvement or a particular task done well (e.g. “time management” or “engaged the audience”); three points were given to a specific comment that identified either a unique area for improvement with a suggestion on how to correct the flaw or a task done well and why it contributed to the strength of the lecture (e.g. “slides were occasionally difficult to read and could be improved by replacing blocks of text with an explanatory bar graph”).

A sample size calculation for the primary aim of presence of comments was performed after 20 evaluation forms were collected with β=.80, and α=.05. Chi-square test assessed presence of comments based on location and Wilcoxon rank-sum test was performed to assess quality of comments. Cohen’s kappa was used to assess agreement of quality score.

### Location of narrative comment section

Upon analyzing 206 evaluation forms, presence and quality of comments did not differ by location of narrative section. Quality of comments also did not differ by location for strengths or weaknesses when analyzed separately. Audience members were more likely to give “strengths” feedback in narrative form than provide “areas for improvement”. Only one respondent gave constructive feedback without a positive comment, whereas 74 respondents gave positive comments without providing a comment for an area of improvement. Reviewers agreed on the quality of comments.

Formative assessment and feedback have been shown to change trainees’ behavior.^2^^-^^5^ In addition to being a requirement of US residency programs, feedback is an important part of gauging lecture effectiveness and an integral part of improving future performance for both experts and trainees alike. Trainees do not benefit from feedback in the form of numerical marks but rather from narrative, specific feedback that explicitly states the areas that require improvement.^3^^,^^6^^,^^8^ We had hypothesized that the frequency and quality of narrative feedback would increase if this portion of the lecture evaluation form were placed at the top, rather than bottom, of the form; however our study demonstrated there was no difference in the frequency or quality of narrative feedback provided. As with other studies in the literature, eliciting high-quality narrative feedback remains a challenge.^9^^,^^10^

## Conclusions

The location of a narrative comment section does not change the presence or quality of comments on a lecture evaluation form. Further investigation is needed to develop a tool to solicit high quality narrative feedback to improve resident physician lecture skills.

### Acknowledgements

The authors would like to thank Patricia Mitchell, Breanne Langlois and Hudson Breaud for their assistance in research design methodology and statistical review.

### Conflict of Interest

The authors declare that they have no conflict of interest.
